# Pressure-Induced Phase Transitions in Bilayer La_3_Ni_2_O_7_

**DOI:** 10.1021/acs.jpcc.5c07252

**Published:** 2025-12-11

**Authors:** Mingyu Xu, Greeshma C. Jose, Aya Rutherford, Haozhe Wang, Stephen Zhang, Robert J. Cava, Haidong Zhou, Wenli Bi, Weiwei Xie

**Affiliations:** 1 Department of Chemistry, 3078 Michigan State University, East Lansing, Michigan 48824, United States; 2 Department of Physics, 9968 University of Alabama at Birmingham, Birmingham, Alabama 35294, United States; 3 Department of Physics and Astronomy, University of Tennessee, Knoxville, Tennessee 37996, United States; 4 Department of Chemistry, 6740 Princeton University, Princeton, New Jersey 08540, United States; 5 Department of Physics and Astronomy, University of South Carolina, Columbia, South Carolina 29208, United States

## Abstract

La_3_Ni_2_O_7_ differs from expectations in part because at ambient pressure, it exists in two polymorphs: an unconventional crystal structure with alternating layers of single- and triple-layered nickel–oxygen octahedra (i.e., La_2_NiO_4_ plus La_4_Ni_3_O_10_, with the so-called 1313 crystal structure) and a classical double-layer Ruddlesden–Popper phase (i.e., La_3_Ni_2_O_7_, with the so-called 2222 crystal structure, which dominates at high pressure). In this study, we report the pressure-dependent structural and electrical resistive properties of single crystals of the classical double-layered La_3_Ni_2_O_7_, grown at slightly elevated pressure using the floating-zone method. Structural characterization under pressures up to 15.4 GPa reveals a gradual transition from orthorhombic to tetragonal symmetry that is completed at 12–14 GPa. Additionally, we present pressure and field-dependent electrical resistance measurements up to 27.4 GPa, from which we construct a phase diagram. Although a resistive transition at 80 K is observed at high pressures, the resistive hallmarks of superconductivity are not.

## Graphical abstract

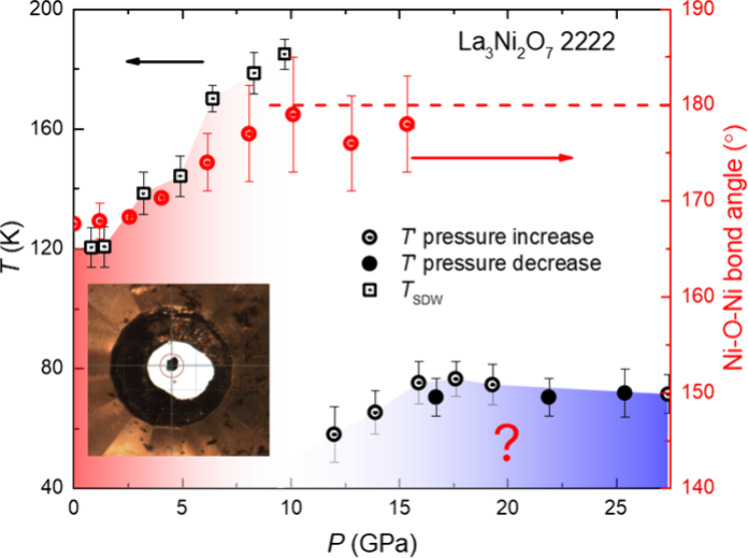

## Introduction

Following the report that La_3_Ni_2_O_7_–_δ_ hosts superconductivity below 80 K at applied pressures of 14–43.5 GPa, significant attention has been focused on nickelate superconductivity, primarily due to the high transition temperatures (*T*_c_) involved and Ni’s proximity in the periodic table to the elements Cu and Fe, which are the well-established basis for high temperature superconductors. Superconductivity has long been a central focus of scientific investigation, particularly since the discovery of high-temperature cuprate superconductors in the 1980s.^1^ The subsequent discovery of Fe-based superconductors,^2,3^ further expanded the field by demonstrating that the presence of usually magnetic ions like Fe^2+^ does not preclude superconductivity, suggesting a connection between magnetism and superconductivity at high temperatures. In this context, nickelates have recently garnered substantial attention.^4–15^ Thin films of the so-called infinite-layer nickelates, with *T*_c_ between 6 and 15 K.^7,14,16^ have been thoroughly studied.^7,8,10,11,17^ More recently, the report of superconductivity in La_3_Ni_2_O_7_–_δ_, with a *T*_c_ of 80 K under applied pressures ranging from 14 to 43.5 GPa^18^ has been seen by many as a major event, as the Tc exceeds the boiling point of liquid nitrogen and also since Ni ions are often magnetic. It therefore appears to satisfy many of the criteria needed for the identification of a new high-temperature superconducting system connecting magnetism and superconductivity.

A surge of research activity has followed the initial report of high-temperature superconductivity in La_3_Ni_2_O_7_, although the limited magnetic shielding fraction, often attributed to filamentary superconductivity near 80 K, remains a central challenge.^19,20^ Evidence for filamentary behavior has been documented in several studies, and the pressure-induced structural evolution has been probed using high-pressure STEM,^20^ TEM, and synchrotron X-ray diffraction.^19,21^ Complementary transport measurements have revealed resistive anomalies across multiple forms of the material, including single-crystalline 1313-La_3_Ni_2_O_7_,^22^ polycrystalline La_3_Ni_2_O_7_,^23^ and Y-doped analogues,^24^ with some liquid-pressure-medium experiments even reporting zero resistance.^25,26^ Spectroscopic techniques such as ARPES and ^139^La NMR have provided further insight into the underlying electronic structure.^27,28^ In particular, La_3_Ni_2_O_7_ has been shown to exhibit strong charge-transfer character, with self-doped holes migrating from Ni to O sites, an aspect that may be intimately related to its superconducting behavior.^29^ Additional magnetic probing using nitrogen-vacancy (NV) centers in diamond has detected a small (2–5%) diamagnetic response.^30^

On the theoretical front, proposed mechanisms for superconductivity in La_3_Ni_2_O_7_ span a wide range. Some studies draw parallels to cuprates, whereas others argue that the pairing mechanism is qualitatively distinct from both cuprates and infinite-layer nickelates.^31,32^ Diverse approaches, including density functional theory, dynamical mean-field theory, and multiorbital modeling, have been used to explore the superconducting state and its origins.^33–36^ Interlayer coupling has been proposed as a key factor enhancing the transition temperature,^37^ and interlayer antiferromagnetic correlations have been shown to promote pairing.^38^ Multiorbital analyses further highlight the importance of the Ni *d*_3*z* ^2^–*r* ^2^_ orbital,^39^ while bilayer Hubbard and effective Hamiltonian models have been constructed to capture the essential physics of the high-pressure superconducting state.^40,41^

Related nickelates show comparably rich behavior. For example, muon spin relaxation measurements have been performed on polycrystalline La_3_Ni_2_O_6.92_ at ambient pressure,^42^ and theoretical investigations of La_3_Ni_2_O_6_ under pressure reveal significant modifications to its electronic and magnetic landscape.^43^ Chemical-pressure tuning has also emerged as a powerful tool: substituting La^3+^ with smaller A^3+^ cations enhances orthorhombic distortions and increases the critical pressure for the tetragonal transition, whereas larger A-site ions (e.g., Sr^2+^, Ba^2+^) appear to stabilize the tetragonal phase more effectively.^44,45^ Superconductivity has been reported to vanish in both ambient- and high-pressure measurements of Sr-doped La_3_Ni_2_O_7_,^46^ though oxygen annealing can improve its conductivity.^47^ Beyond La-based compounds, bulk superconductivity has been claimed in La_2_PrNi_2_O_7_,^48^ and first-principles calculations predict pressure-driven electronic reconstructions in *A*_3_Ni_2_O_7_ (*A* = Nd–Lu, Y, Sc), with Tb_3_Ni_2_O_7_ suggested as a promising ambient-pressure superconducting candidate.^49^ Low-volume-fraction superconductivity has also been observed in Pr_4_Ni_3_O_10_ under pressure,^50^ and hybrid Ruddlesden–Popper nickelates have recently been synthesized and characterized, further expanding the chemical and structural landscape of nickelate superconductivity.^51^

At ambient pressure, La_3_Ni_2_O_7_ exists in two distinct polymorphs: one based on alternating single- and triple-layered nickel–oxygen octahedral layers (the 1313 phase, space group *Cmmm*), and the other featuring double-layered nickel–oxygen octahedra in a classical Ruddlesden–Popper phase, (The more conventional 2222 phase, space group *Cmcm*).^52,53^ While La_3_Ni_2_O_7_ in its 1313 form has been proposed as the potential host phase for the high-pressure superconductivity,^19^ and thus is widely studied, the high-pressure behavior of the 2222 phase, the subject of this study, remains relatively unexplored. In this work, we characterized La_3_Ni_2_O_7_-2222 single crystals grown using the floating zone method. The single crystals were characterized through single-crystal X-ray diffraction and electrical resistance measurements at various pressures. A pressure–temperature phase diagram is constructed based on these measurements, revealing a continuous structural transition that is complete at about 12–14 GPa. This high-pressure phase appears to show a transition near 80 K in the resistivity that has none of the hallmarks that have been attributed to superconductivity but is reminiscent of the “filamentary superconductivity” claimed by others.^19,20^

## Experimental Details

### Single Crystal Growth of La_3_Ni_2_O_7_

Single crystals of La_3_Ni_2_O_7_-2222 were grown using the floating zone method in a vertical optical-image furnace. Stoichiometric mixtures of La_2_O_3_ (pretreated at 1000 °C to remove H_2_O) and NiO were thoroughly ground and fired at 1050 °C for 24 h. The precursor powders were then hydrostatically pressed into rods and sintered at 1400 °C for 12–24 h. Crystal growth was performed directly from the sintered rods under a 100% O_2_ atmosphere at approximately 14–15 bar pressure. During growth, the traveling rate of the crystal boule was maintained at 3–4 mm/h, with the feed rod and seed counter-rotated at 15–20 rpm. The La_3_Ni_2_O_7_-2222 crystals were successfully extracted from the resulting boule.

### Crystal Structure

A single crystal with approximate dimensions of 0.069 × 0.045 × 0.014 mm^3^ was chosen for structural determination. The crystal was mounted on a nylon loop with Paratone oil and examined at room temperature using a Rigaku XtalAB Synergy Dualflex diffractometer equipped with Mo Kα radiation (λ = 0.71073 Å, 50 kV, 1 mA). Diffraction frames were collected via ω-scans, and data-acquisition strategies, including frame count, exposure distribution, and detector positioning, were automatically optimized within the CrysAlisPro suite (version 1.171.42.101a). Raw data were corrected for Lorentz and polarization effects and subsequently underwent numerical absorption correction based on a multifaced Gaussian integration routine.^54^ Additional empirical absorption corrections using spherical harmonics were applied via the SCALE3 ABSPACK module to improve scaling quality further.^55^ Structure solution and refinement were performed using the SHELXTL package in the orthorhombic space group *Cmcm*, with Z = 4.^56,57^ No reflections indicative of a 1313-type intergrowth was observed, suggesting that either the synthetic conditions or the oxygen fugacity favor the formation of the conventional 2222-phase.

### High-Pressure Single-Crystal X-ray Diffraction

The X-ray diffraction (XRD) experiments at applied pressures up to 15.4 GPa were carried out at the identical single crystal specimen of La_3_Ni_2_O_7_-2222 that was characterized at ambient pressure. For the high-pressure measurements, the sample was loaded into a Diacell One20DAC (Diamond Anvil Cell) manufactured by Almax-easyLab, equipped with 500 μm culet-size Boehler-Almax type anvils. A 250 μm thick stainless-steel gasket was preindented to 48 μm. After that, a 210 μm hole was drilled using an electric discharge machining system (EDM) to accommodate the sample. The single crystal was positioned with its largest facet approximately perpendicular to the incident X-ray beam to optimize reflection intensities. Ruby spheres were placed adjacent to the sample for in situ pressure calibration. A 4:1 methanol–ethanol mixture was employed as the pressure-transmitting medium to ensure that hydrostatic conditions were operative^58^ up to about 10 GPa and minimize deviatoric stresses at higher pressure. The pressure inside the cell was monitored using the *R*_1_ fluorescence line of a ruby, ensuring accurate pressure measurements.^59–61^

### High-Pressure Electrical Resistance Measurements

The high-pressure electrical resistance measurements in the basal plane of the conventional 2222-structure La_3_Ni_2_O_7_ single crystal were conducted in a Quantum Design Physical Property Measurement System (PPMS-DynaCool). The Electrical Transport Option (ETO) option was used for the four-probe electrical resistance measurements, which were performed in the standard van der Pauaw geometry using thin Pt foils as electrodes. High pressure was achieved using a pair of diamond anvils of 500 μm diameter culet size in a diamond anvil cell made of Be–Cu alloy. Two experimental runs were conducted using AC currents of 0.5 mA at a frequency of 18.3 Hz on two single crystals; no change was observed at lower currents. A stainless-steel gasket, initially 250 μm thick, was compressed to a thickness of 75 μm, and a 176 μm diameter hole was drilled in its center. To insulate the electrode leads from the metallic gasket, the gasket was coated with a fine mixture of cubic boron nitride powder and epoxy. The La_3_Ni_2_O_7_ single crystal was placed in center of the gasket hole. Pressures were applied at room temperature and measured by the ruby fluorescence method.^62^ NaCl pressure medium is used in this experiment.

## Results and Discussion

Figure 1a illustrates the crystal structures of La_3_Ni_2_O_7_-2222 at both ambient pressure and 15.4 GPa. With the exception of a change from orthorhombic to tetragonal symmetry, commonly observed in *I*4/*mmm* Ruddelden-Popper phases,^52,53^ the basic crystal structure of our 2222 single crystal is maintained in this pressure range. [Detailed information regarding the ambient pressure X-ray diffraction measurements is provided in the Supporting Information (Tables SI and SII)]. La_3_Ni_2_O_7_-2222 has a classical Ruddlesden–Popper bilayer stacking, featuring a pseudo-F-centered orthorhombic lattice with its double NiO2 layers separated by rock salt [LaO] layers.^53^ As depicted in Figure 1c, the lattice parameters and unit cell volume decrease with increasing pressure, and, as shown in Figure 1a,d, the NiO6 octahedra gradually untwist. At ambient pressure, the Ni–O1–Ni bond angle is approximately 167.8° and as pressure increases to about 12 GPa, this bond angle approaches 180°, while the ratio of the *c* to *b* lattice parameters (*c*/*b*) approaches 1. This behavior indicates a smooth structural transformation from the orthorhombic to the tetragonal phase. Within our experimental resolution, the transition is consistent with a second-order process, as evidenced by the continuous evolution of the lattice parameters without any abrupt changes (Figure 1c). Furthermore, the absence of any volume discontinuity or collapse across the transition pressure (Figure 1e) aligns with the thermodynamic criteria for a continuous, second-order structural transition. Reciprocal-space reconstructions from our high-pressure single-crystal diffraction data also reveal no coexistence of orthorhombic and tetragonal reflections, providing additional support for the continuous nature of the symmetry change. The volume change as a function of pressure is shown in Figure 1e with the Birch–Murnaghan fit yielding B_0_ = 143.6 GPa.

$$P\left(V\right)=3\frac{B_{0}}{2}\left({\left(\frac{V_{0}}{V}\right)}^{7/3}-{\left(\frac{V_{0}}{V}\right)}^{5/3}\right)\left(1+\left(\frac{3}{4}\right)\left(B_{0}^{\prime }-4\right)\left({\left(\frac{V_{0}}{V}\right)}^{2/3}-1\right)\right)$$

**Figure 1:**
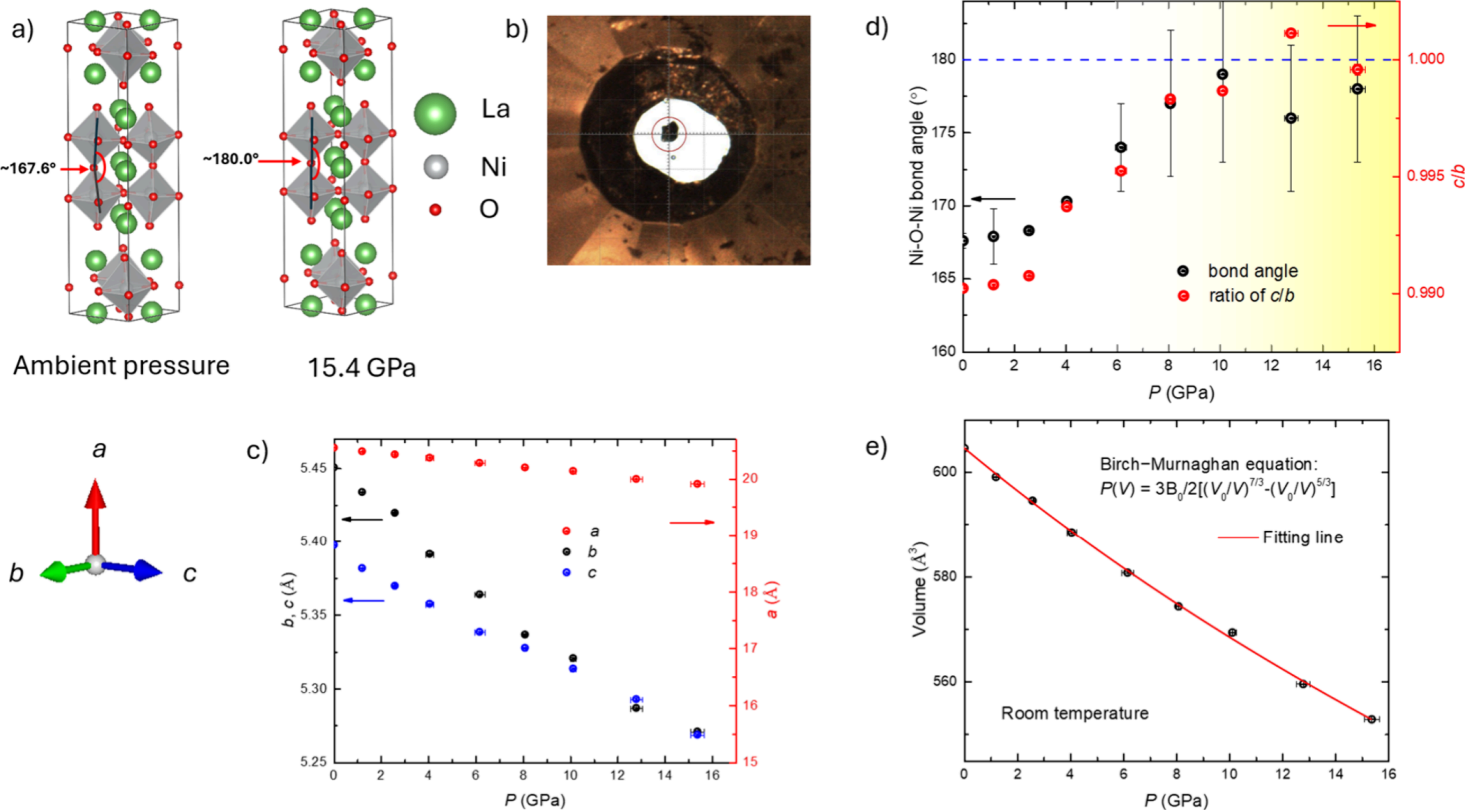
Crystal structure, lattice parameter, and bond angle information for single crystal La_3_Ni_2_O_7_-2222 under Pressure up to 15.4 GPa. (a) Crystal structure of La_3_Ni_2_O_7_-2222, at 0 and 15.4 GPa. (Visualized using VESTA software.^63^) (b) The sample was studied inside the Diamond Anvil Cell (DAC) at a pressure of 15.4 GPa. (c) The lattice parameters (*a*, *b*, and *c*) as a function of pressure. (d) The Ni–O1–Ni bond angle and the ratio of the *c* to *b* lattice parameters (*c*/*b*) under pressure. The blue dashed line marks a Ni–O1–Ni bond angle of 180° and a *c*/*b* ratio of 1. The yellow-shaded region highlights the pressure range where the change from orthorhombic to tetragonal symmetry has occurred. (e) The volume change as a function of pressure with the Birch–Murnaghan fit shown.

*V*_0_ is zero-pressure volume, *V* is volume at pressure *P*, *B*_0_ is Bulk modulus at zero-pressure, and *B*’_0_ is pressure derivative of the bulk modulus.

Figure 2a,b display the temperature dependence of the electrical resistance of a La_3_Ni_2_O_7_-2222 single crystal in different pressure regimes. In the low-pressure range, the material is highly insulating, though a kink-like feature appears on cooling at around 130 K, accompanied by a broad hump near 220 K, consistent with measurements reported by other groups.^23^ These kink-like features have been attributed to spin density wave transitions (T_SDW_) by others.^23,64–67^ As the pressure rises, these features become progressively weaker and are eventually suppressed, and the material becomes more conducting. In the pressure range below 12 GPa, the electrical resistance as a function of temperature exhibits insulating behavior over the entire temperature range studied. However, as pressure increases, the resistance–temperature relationship shifts toward more metallic character. By 19.3 GPa, the metallic behavior of the resistance becomes evident. At pressures above 12–14 GPa, a pressure where the symmetry of La_3_Ni_2_O_7‑_2222 is tetragonal, another, lower temperature feature first emerges, becomes more pronounced, and then stabilizes near 80 K. This looks like the feature that has been attributed to superconductivity,^18^ although unfortunately, the resistance does not drop to 0 and the resistive transition is very broad.

**Figure 2:**
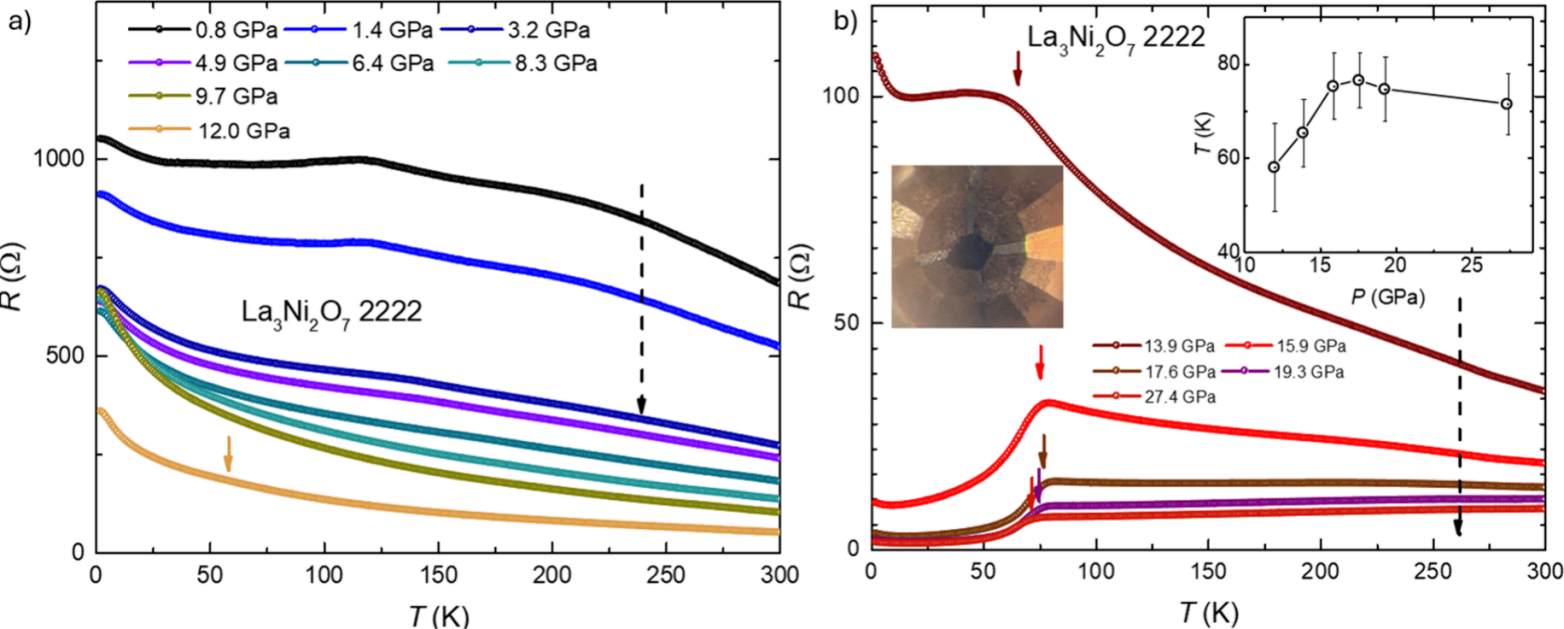
Electrical transport measurements of a La_3_Ni_2_O_7_-2222 single crystal at pressures up to 27.4 GPa. (*a*). The electrical resistance of La_3_Ni_2_O_7_-2222 as a function of temperature (from 2 to 300 K) at pressures up to 12.0 GPa. The black dashed line indicates the direction of increasing pressure, while the colored arrows mark the transition temperatures. (*b*). The electrical resistance versus temperature in the pressure range of 13.9 to 27.4 GPa. The inset shows a picture of the sample in the DAC. The right inset shows the evolution of transition as a function of pressure.

Figure 3a presents the pressure–temperature (P-T) phase diagram of La_3_Ni_2_O_7_-2222, based on the criteria outlined in the Supporting Information (Figure S1a,b). The transition temperatures for what has been identified as a spin density wave transition (T_SDW_) and the low-temperature transition (T’) were determined resistively from the mean values of the onset and transition completion temperatures. The error bars represent the transition widths defined in Figure S1a,b. T_SDW_ increases with increasing pressure, consistent with recent muon-spin rotation/relaxation (μSR) studies.^66^ (The transition temperatures observed in both types of experiments are comparable, with minor differences likely attributed to variations in measurement criteria and sample heterogeneity.) The spin density wave vanishes at pressures near 12 GPa - the pressure at which the Ni–O1–Ni bond angle reaches 180°, marking a structural transition from orthorhombic to tetragonal symmetry. Another resistive transition (here called T’) emerges at approximately the same pressure. T’ stabilizes at approximately 80 K after 15.9 GPa and remains nearly constant in temperature as the pressure increases up to 27.4 GPa, the limit of our resistance measurements. Finally, Figure 3b shows the electrical resistance at 300 K as a function of pressure, showing how R­(300 K) decreases significantly as the pressure increases.

**Figure 3:**
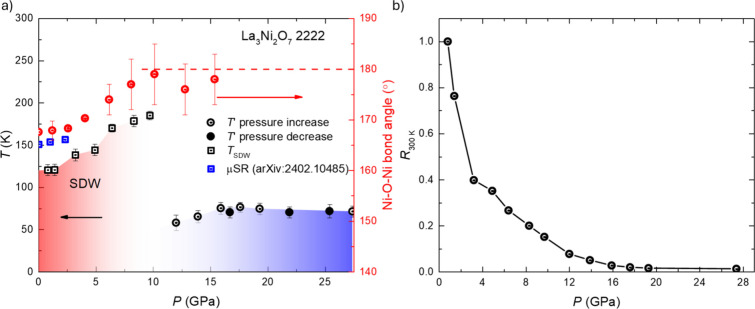
Pressure–temperature (P-T) phase diagram and the resistance at 300 K of a single crystal of 2222-La_3_Ni_2_O_7_. (a). The P-T phase diagram for La_3_Ni_2_O_7_ −2222 based in part on our electrical resistance and structural characterization. The black squares represent the spin density wave transition temperature (T_SDW_) determined by high-pressure resistance measurements, while the blue square gives the results of μSR studies.^66^ On the right-hand side of Figure 3a, the Ni–O1–Ni bond angles are plotted using red symbols, with the red dashed line indicating the 180° bond angle. The black hollow circles indicate the transition temperature (T’), while the black solid circles show T’ during pressure release (b). The electrical resistance at 300 K as a function of pressure for La_3_Ni_2_O_7_-2222.

To determine whether there is a significant applied magnetic field dependence to the 80 K transition, as is seen for superconductors, we measured the resistance in magnetic fields. Figure 4a illustrates the temperature dependence at 27.4 GPa of the normalized resistance (*R*(T)/*R*(150 K)) of our La_3_Ni_2_O_7_-2222 single crystal in applied magnetic fields between 0 and 90 kOe (0 to 9 T). The sudden drop in resistance near 80 K that has been attributed to superconductivity^18^ studying La_3_Ni_2_O_7_; which we observe in a classical 2222 La3Ni2O7 single crystal, does not change much with applied magnetic field. The inset emphasizes this observation with Figure 4b showing the corresponding temperature-field behavior. Both figures demonstrate that the onset values, even using different criteria, of the transition shift only slightly in response to the applied magnetic field. No clear magnetic-field-induced suppression of the transition temperature is observed, unlike the pronounced field sensitivity seen in YBa_2_Cu_3_O_7‑δ_ (Figure 4b and Figure S1c), which would ordinarily be expected for a superconducting transition. Nevertheless, the present results do not fully exclude the presence of filamentary superconductivity. If superconductivity is confined to an extremely small volume fraction, consistent with the absence of zero resistance, its contribution to the total resistivity would be negligible and therefore unlikely to exhibit a measurable field dependence. Although the magnetic field does not significantly influence the transition temperature or the resistance prior to the transition, a noticeable change in resistance occurs under the applied magnetic fields, below the onset, the transition broadens. This also occurs in some antiferromagnetic systems such as Nd_3_TiSb_5_.^68^ Similar temperature-dependent resistance behavior under different magnetic fields was observed at 15.9 GPa (Figure S2), further supporting these findings. Together, these results suggest that a magnetic transition, potentially due to the elimination of the magnetic scattering from a spin density wave, exists at around 80 K for La_3_Ni_2_O_7_-2222 in the high-pressure range (above 12 GPa), or, based on the literature, that this is a filamentary superconducting transition.

**Figure 4:**
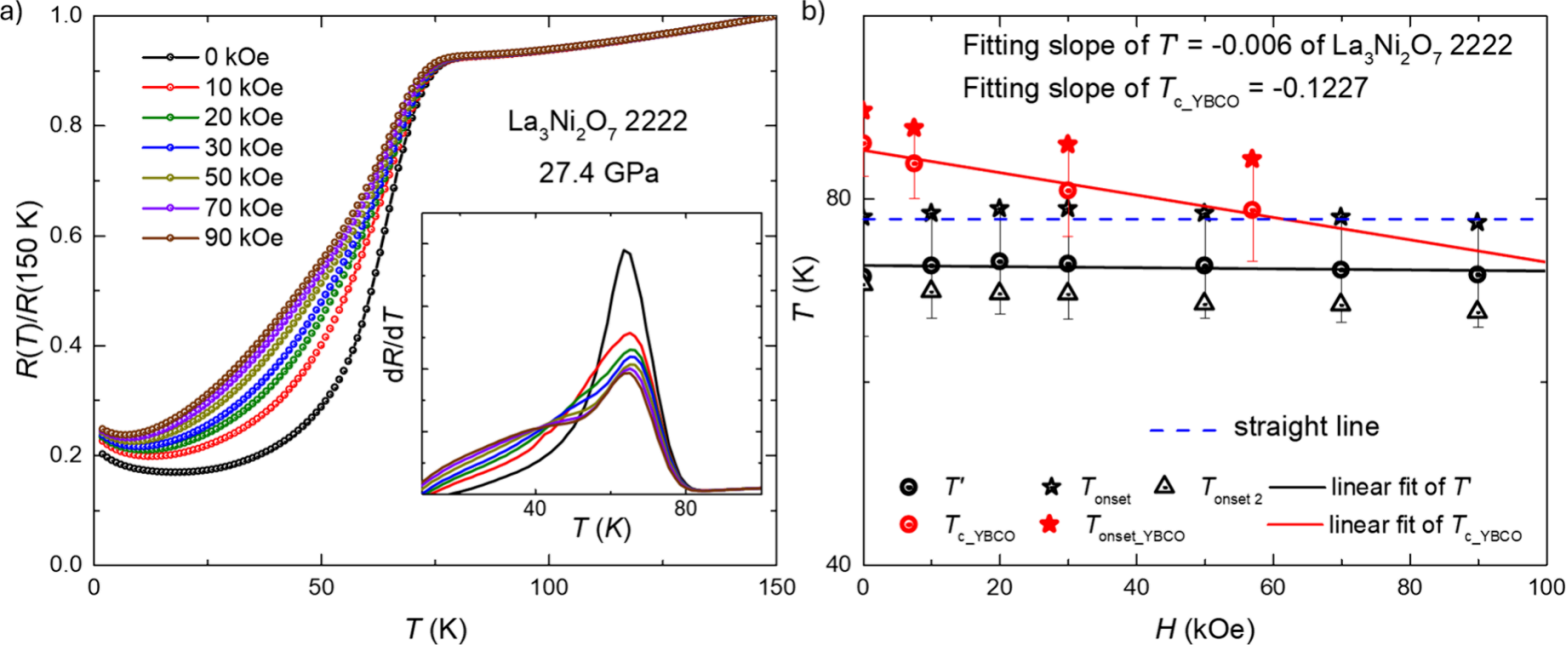
Normalized resistance under pressure for different magnetic fields. (a) Detail of the normalized resistance (*R*(*T*)/*R*(150 K)) of La_3_Ni_2_O_7_-2222 under different magnetic fields and (b) the *H*-*T* behavior of the 80 K transition in La_3_Ni_2_O_7_-2222 at 27.4 GPa. In (b) The black circle symbols show the transition temperatures determined using the criterion in Figure S2, the T_onset_ is the onset value. T_onset 2_ is given by a different criterion, which is 90% of *R* (77.1 K), which is defined as the highest value before the large drop. The red symbols give the results for YBCO, with nearly the same Tc, using the same criteria. The data for YBCO is digitized from reference.^69^ (Copyright from American Physical Society with the license number: RNP/25/NOV/099315.)

## Conclusion

La_3_Ni_2_O_7_-2222 single crystals were grown at a slightly enhanced oxygen pressure using the floating zone method and subjected to high-pressure single-crystal X-ray diffraction and electrical resistance measurements. These experiments allowed for the construction of a temperature–pressure phase diagram. The ambient pressure phase is stable up to about 12 GPa, consistent with previous μSR results.^66^ At about 12 GPa, a structural transition from orthorhombic to tetragonal symmetry is observed in La_3_Ni_2_O_7_-2222, accompanied by an 80 K phase transition. This transition does not exhibit the characteristics typical of superconductivity, as the transition temperature remains largely unaffected by an applied magnetic field, the material displays insulating or semiconducting behavior prior to the transition, and the transition is to a nonzero resistance state. Given the significant resistance drop and the presence of an SDW, we speculate that this transition is likely a magnetic transition that reduces the magnetic scattering or another a filamentary superconducting transition. We specifically point out that the temperature of the 80 K transition is only slightly changed in the range of pressures between about 15 and 25 GPa, which, along with the independence of the magnetic field, suggests that strong magnetic forces are involved. Further experiments are clearly required to determine the detailed nature of the high-pressure La_3_Ni_2_O_7_-2222 phase below 80 K.

## Supplementary Material





## Notes

Atomic coordinates and isotropic displacement parameters; criterion of T_DW_ and T′ based on the plots of d*R*/d*T* at different pressures; field-dependent resistivity of La_3_Ni_2_O_7_-2222 at 15.9 GPa (PDF)
Crystal structure refinement data (ZIP)
