# Supplementary material for: Pressure-Induced Phase Transitions in Bilayer La3Ni2O7
Source: J Phys Chem C Nanomater Interfaces. 2025 Dec 11;130(13):4911–8. doi: 10.1021/acs.jpcc.5c07252 (PMC13051444; doi:10.1021/acs.jpcc.5c07252)
Supplement: Supplementary file 1 [file jp5c07252_si_001.pdf]

Supporting Information

Pressure-Induced Phase Transitions in Bilayer $La_3Ni_2O_7$

Mingyu Xu[1], Greeshma C. Jose[2], Aya Rutherford[3], Haozhe Wang[1], Stephen Zhang[4], Robert J. Cava[4], Haidong Zhou[3], Wenli Bi[5], Weiwei Xie[1*]

[1]Department of Chemistry, Michigan State University, East Lansing, MI 48824, USA
[2]Department of Physics, University of Alabama at Birmingham, Birmingham, AL 35294, USA
[3]Department of Physics and Astronomy, University of Tennessee, Knoxville, TN 37996, USA
[4]Department of Chemistry, Princeton University, Princeton, NJ 08540, USA
[5]Department of Physics and Astronomy, University of South Carolina, Columbia, SC 29208 USA

Corresponding author: Weiwei Xie (xieweiwe@msu.edu)

Table of Content

Table SI. Crystal structure refinement data…………………………………………………...S2

Table SII. Atomic coordinates and isotropic displacement parameters……...............................S2

Fig. S1. Criterion of T_{DW} and T' based on the plots of d*R*/d*T* at different pressures……..… S4

Fig. S2. The field dependent resistivity of La3Ni2O7-2222 at 15,9 GPa……………………S5

Table SI. The crystal structure and refinement of $La_3Ni_2O_7$ at room temperature and ambient pressure (Mo Kα radiation). Values in parentheses are estimated standard deviations from the refinement.

Chemical Formula	$La_3Ni_2O_7$
Formula Weight	646.15 g/mol
Space Group	*Cmcm*
Unit Cell dimensions	a = 20.5510(13) Å<br>b = 5.4508(4) Å<br>c = 5.3975(4) Å
Volume	604.62(7) $Å^3$
Z	4
Density (calculated)	7.098 g/cm^3
Absorption coefficient	26.849 mm^{-1}
F (000)	1132
2θ range	7.734 to 81.316°
Reflections collected	4856
Independent reflections	1047 [R_{int} = 0.0701]
Refinement method	Full-matrix least-squares on F^2
Data/restraints/parameters	1047/0/37
Final R indices	R_1 (I>2σ(I)) = 0.0460; wR_2 (I > 2 σ(I)) = 0.1067<br>R_1 (all) = 0.0862; wR_2 (all) = 0.1261
Largest diff. peak and hole	+7.82 $e^-/Å^3$ and -2.74 $e^-/Å^3$
R. M. S. deviation from mean	0.769 $e^-/Å^3$
Goodness-of-fit on F^2	0.997

Table SII. Atomic coordinates and equivalent isotropic atomic displacement parameters ($Å^2$) of $La_3Ni_2O_7$ at ambient pressure and 300 K. (U_{eq} is defined as one-third of the trace of the orthogonalized U_{ij} tensor.) Values in parentheses are estimated standard deviations from refinement.

Atoms	Wyck.	*x*	*y*	*z*	Occ.	U_{eq}
**La1**	4*c*	0	0.75046(11)	1/4	1	0.008(14)
**La2**	8*g*	0.32020(2)	0.25758(7)	1/4	1	0.007(13)
**Ni**	8*g*	0.09594(5)	0.25201(16)	1/4	1	0.005(19)
**O1**	4*c*	0	0.29120(15)	1/4	1	0.008(15)
**O2**	8*g*	0.20360(3)	0.21760(12)	1/4	1	0.012(13)
**O3**	8*e*	0.39500(4)	0	0	1	0.013(13)
**O4**	8*e*	0.08990(3)	0	0	1	0.012(14)

Tables SI and SII show the results of the single-crystal XRD. The structure was solved and refined using the Bruker SHELXTL Software Package with the space group *Cmcm*, $La_3Ni_2O_7$. The final anisotropic full-matrix least-squares refinement on F^2 with 37 variables converged at R_1 = 8.62%, for the observed data and wR_2 = 12.61% for all data. The goodness-of-fit was 0.997. The largest peak in the final difference electron density synthesis was 7.82 $e^-/Å^3$, and the largest hole was -2.74 $e^-/Å^3$ with an RMS deviation of 0.769 $e^-/Å^3$. Based on the final model, the calculated density was 7.098 g/cm^3 and F (000), 1132 e^-.

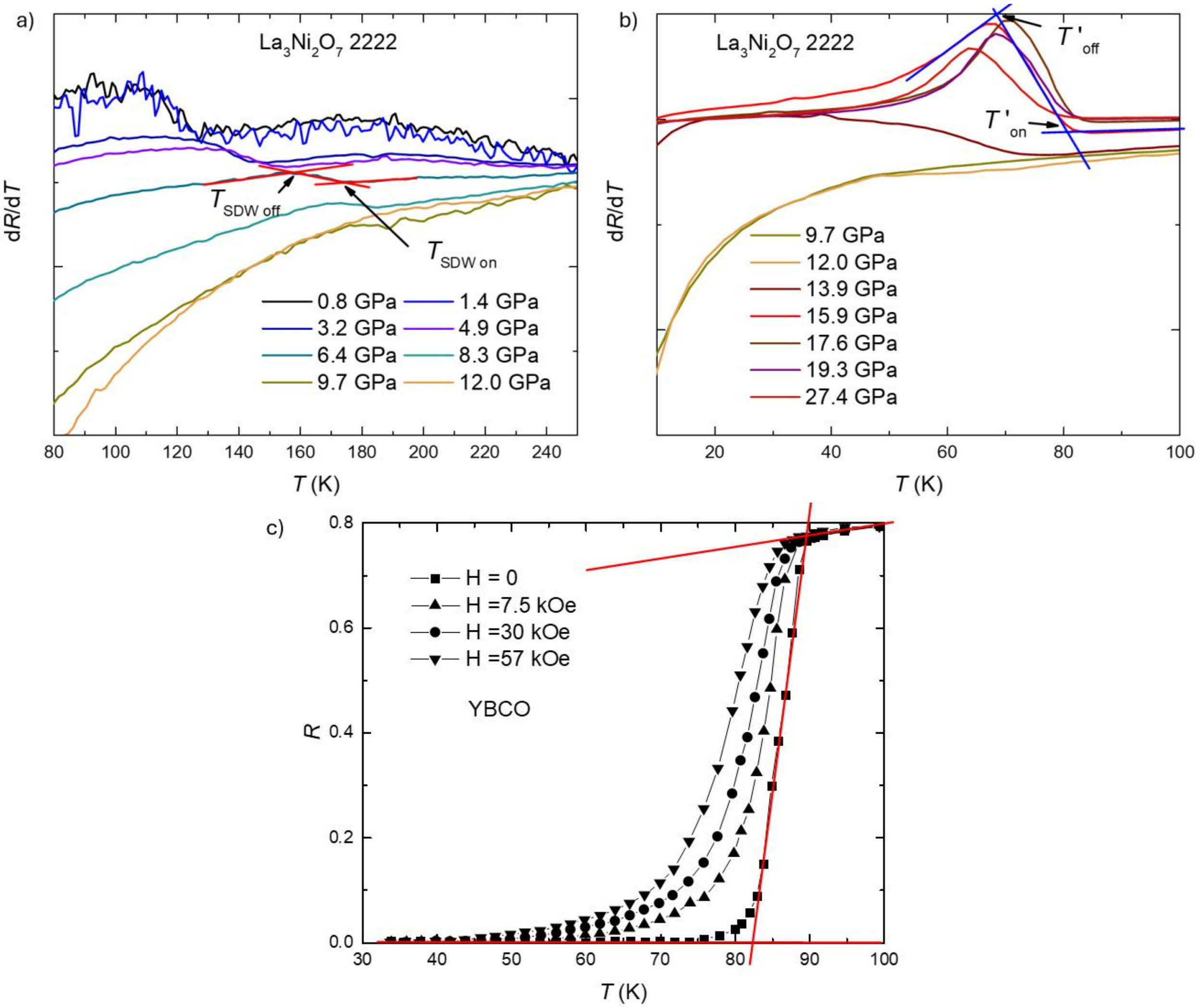

Fig. S1. Criterion of T_{DW} and T' based on the plots of d*R*/d*T* at different pressures. Fig. S1*a* shows the criterion (onset and offset value) of T_{SDW} using d*R*/d*T* in the temperature range from 80 K to 150 K. **Fig. S1*b*** gives the criterion of T' based on d*R*/d*T* in the temperature range from 10 K to 100 K. **Fig. S1*c*** gives digitized data from M. K. Wu, J. R. Ashburn, and C. J. Torng, P. H. Hor, R. L. Meng, L. Gao, Z. J. Huang, Y. Q. Wang, and C. W. Chu, Superconductivity at 93 K in a new mixed-phase Y-Ba-Cu-O compound system at ambient pressure, Phys. Rev. Lett. **58**, 908 (1987).

Fig. S1 shows the criteria for the determination of T_{SDW} and *T*'. The values of T_{SDW} and *T*' are given by the mean values of onset and offset, and the transition width is given by the half value of the difference of onset and offset.

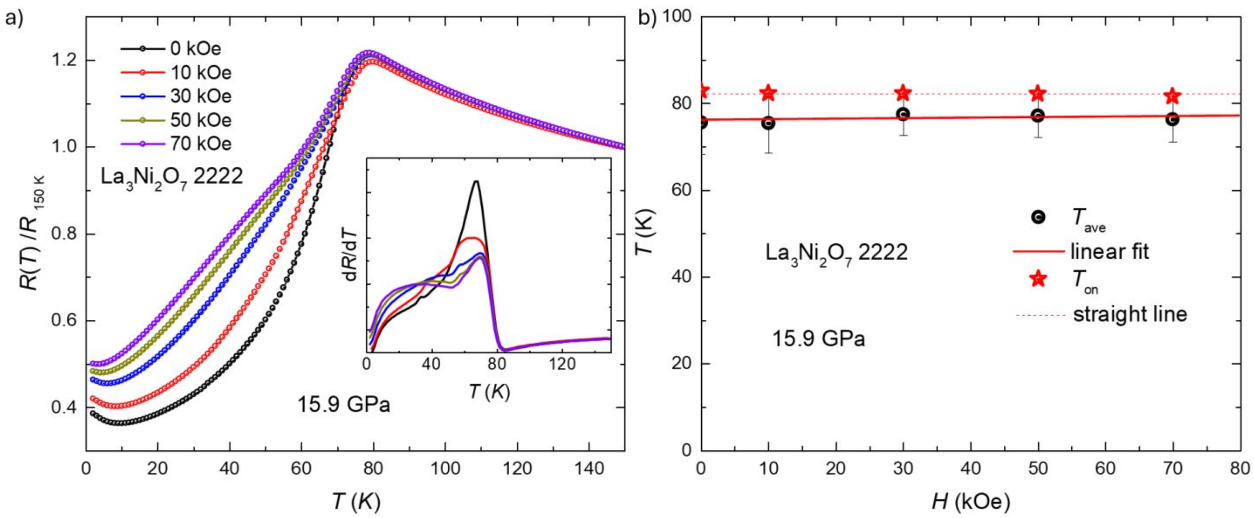

Fig. S2. Normalized resistance (*R*(*T*)/*R*(150 K)) of $La_3Ni_2O_7$-2222 under the different magnetic fields (Fig. S2*a*) and *T*'**-*H* phase diagram (Fig. S2*b*) at 15.9 GPa. Fig. S2*a*** shows the normalized resistance as a function of temperature under the different magnetic fields at 15.9 GPa. (Inset) The derivative of resistance as a function of temperature. **Fig. S2*b*** shows the temperature-magnetic field (*T*-*H*) phase diagram. The black symbols show the *T***'** values, and the red star symbols present the onset values. The red solid line gives the linear fit of *T***'**, and the red dashed line presents the straight line across the onset values.
